# Planning for health equity in the Americas: an analysis of national health plans

**DOI:** 10.26633/RPSP.2021.29

**Published:** 2021-04-28

**Authors:** Matthew M. Kavanagh, Laura Fernanda Norato, Eric A. Friedman, Adria N. Armbrister

**Affiliations:** 1 Georgetown University Washington D.C. United States of America Georgetown University, Washington D.C., United States of America; 2 Pan American Health Organization Washington D.C. United States of America Pan American Health Organization, Washington D.C., United States of America

**Keywords:** Health equity, public policy, health policy, health systems plans, Americas, Equidad en salud, política pública, política de salud, planes de sistemas de salud, Américas, Equidade em saúde, política pública, política de saúde, planos de sistemas de saúde, América

## Abstract

There is growing recognition that health and well-being improvements have not been shared across populations in the Americas. This article analyzes 32 national health sector policies, strategies, and plans across 10 different areas of health equity to understand, from one perspective, how equity is being addressed in the region. It finds significant variation in the substance and structure of how the health plans handle the issue. Nearly all countries explicitly include health equity as a clear goal, and most address the social determinants of health. Participatory processes documented in the development of these plans range from none to extensive and robust. Substantive equity-focused policies, such as those to improve physical accessibility of health care and increase affordable access to medicines, are included in many plans, though no country includes all aspects examined. Countries identify marginalized populations in their plans, though only a quarter specifically identify Afro-descendants and more than half do not address Indigenous people, including countries with large Indigenous populations. Four include attention to migrants. Despite health equity goals and data on baseline inequities, fewer than half of countries include time-bound targets on reducing absolute or relative health inequalities. Clear accountability mechanisms such as education, reporting, or rights-enforcement mechanisms in plans are rare. The nearly unanimous commitment across countries of the Americas to equity in health provides an important opportunity. Learning from the most robust equity-focused plans could provide a road map for efforts to translate broad goals into time-bound targets and eventually to increasing equity.

## RESUMEN

Cada vez es mayor el reconocimiento de que las mejoras en cuanto a la salud y el bienestar no han llegado por igual a todos los segmentos de la población en la Región de las Américas. En este artículo se analizan 32 políticas, estrategias y planes nacionales del sector de la salud con respecto a diez áreas distintas relativas a la equidad en la salud. El objetivo es comprender, desde una perspectiva, cómo se está abordando la equidad en la Región. Se ha encontrado una variación significativa, tanto en sustancia como en estructura, sobre la manera en que se maneja el tema en los planes de salud. Casi todos los países incluyen explícitamente la equidad en la salud como una meta clara y la mayoría abordan los determinantes sociales de la salud. En la formulación de estos planes se ha documentado desde ningún proceso participativo hasta procesos participativos exhaustivos y sólidos. En muchos planes se han incluido políticas sustantivas centradas en la equidad, como aquellas para mejorar la accesibilidad física a la atención de salud y el acceso a medicamentos asequibles, pero en ningún país se incorporan todos los aspectos analizados. Si bien los países contemplan a los grupos marginados en sus planes, solo una cuarta parte identifica específicamente a las personas afrodescendientes y más de la mitad de los países no considera a las personas indígenas, incluso en el caso de algunos países con una población indígena grande. Cuatro países contemplan la atención médica a los migrantes. A pesar de que existen metas sobre la equidad en la salud y datos de línea de base sobre las inequidades, menos de la mitad de los países incluyen metas con plazos para reducir las inequidades en la salud absolutas o relativas. No son habituales tampoco en los planes los mecanismos de rendición de cuentas claros, como educación, presentación de informes o cumplimiento de los derechos. Los países de la Región de las Américas muestran un compromiso casi unánime con la equidad en la salud, lo cual brinda una oportunidad importante. Aprender de los planes para la equidad más sólidos podría proporcionar una hoja de ruta para las iniciativas que tratan de traducir algunas metas amplias en metas con plazos específicos que puedan eventualmente mejorar la equidad.

## RESUMO

É cada vez mais aceito que os avanços em saúde e bem-estar não são partilhados por todas as populações nas Américas. Neste artigo são analisadas 32 políticas, estratégias e planos nacionais de saúde em 10 áreas distintas de equidade em saúde com o objetivo de entender, de uma única perspectiva, como a equidade está sendo contemplada na região. Existem diferenças consideráveis na forma e conteúdo do enfoque dado a esta questão nos planos de saúde. Quase todos os países estabelecem explicitamente a equidade em saúde como uma meta clara e a maioria aborda os determinantes sociais da saúde. O processo participativo documentado na elaboração dos planos também é variável, desde inexistente a amplo e decidido. Muitos planos contêm políticas concretas com foco central em equidade, por exemplo, políticas para melhorar a acessibilidade física à assistência de saúde e o acesso a medicamentos a preços razoáveis, mas nenhum país inclui todos os aspectos aqui examinados. Os países identificam as populações marginalizadas nos seus planos, porém, apenas um quarto distingue especificamente os afrodescendentes e mais da metade não contempla os povos indígenas, mesmo onde as populações indígenas são em grande número. Quatro países consideram a atenção aos migrantes. Embora existam metas de equidade em saúde e dados relativos a iniquidades de base, menos da metade dos países incorpora em seus planos metas com prazos definidos para reduzir as desigualdades absolutas ou relativas em saúde. Instrumentos claros de responsabilidade como educação, prestação de contas ou respeito aos direitos são raramente vistos. O compromisso praticamente unânime dos países das Américas com a equidade em saúde oferece uma oportunidade importante. Os planos mais bem fundados com enfoque em equidade poderiam servir de exemplo para guiar os esforços de converter metas gerais em metas com prazos definidos e, em última instância, aumentar a equidade.

There is a growing recognition that improvements in health and well-being have not been shared across populations. Among world regions, the Americas have a disproportionate share of highly unequal contexts, as measured in terms of income inequality (1), access to health care, and well-being (2–7). Empirical evidence suggests a central role for public policy in producing or shifting the drivers of inequity (8). There is also some evidence that targeted national policies have improved disparities in access and use of health services in the Americas (9–11). However, there is little agreement on whether good policy planning or effective programming is responsible for the documented advances. Recent assessments of health equity in policy have treated proper planning for equity and the execution of pro-equity interventions together as a single activity, while others argue that proper agenda-setting without implementation can actually widen health inequalities (12–14). Still others posit that the formulation of sound, evidence-based health policy is a requirement for the achievement of health equity (14–16). A strong conceptual framework has recently been given to health equity in the Americas and other regions, which is newly enabling a type of explicit planning and strategizing to reduce inequities in health (17, 18).

This article addresses a core question for health equity: are governments in the Americas planning robustly to address health equity? The striking inequities in health could be a reflection of strong national plans that have not been able to be implemented or whose strategies to address inequity have not been successful. Alternatively, it could be that countries are not planning robustly for health equity at a national level. These two different contexts would suggest quite different paths for international and national decisionmakers seeking to drive more equitable health in the region. This question is particularly acute in the context of the Commission of the Pan American Health Organization (PAHO) on Equity and Health Inequalities in the Americas, which called on countries to “make health equity a key indicator of societal development and establish mechanisms of accountability,” including planning for health equity (19). Are countries already doing so? Proceeding from the premise that such policy planning has a central role in reducing health inequalities, this article provides a situation analysis of the integration of health equity into health plans in the Americas and alignment with key goals articulated in the PAHO Equity Commission report. This analysis employs a rubric meant to give insight into the inclusion of health equity in 32 national health plans along with a series of 10 categories that define priority actions toward equity in health.

There is significant variation in the substance and structure of how the health plans incorporate health equity. This analysis shows that some areas and issues of health equity are tackled far more widely and robustly, while others are addressed by very few countries, with no clear pattern by gross domestic product (GDP) or geography about how countries address health equity. Most of the plans assessed do include the term “health equity” as part of the document’s mission or vision and display a strong focus on the social determinants of health. However, most lack specific measurements to assess progress on addressing inequalities and accountability mechanisms to achieve health equity results. Intentions to address discrimination as a driver of health inequalities are less prevalent in plans than expected.

The overall objective of this paper is to assess the degree to which written national health plans for countries in the Americas plan explicitly for addressing health equity.

## MATERIALS AND METHODS

Based on a review of literature and practice in health equity (16, 20), a rubric was developed to code and analyze health policy environments’ inclusion of health equity. This rubric draws explicitly on the analytic framework of the PAHO Equity Commission (19, 21) and work on health equity programs of action, which have been proposed as a systematic approach to address health equity (20). Starting with these frameworks, a comprehensive but manageable set of 31 indicators (for a total of 43 questions when sub-questions are included) across 10 domains was selected: 1) mission; 2) social and environmental determinants of health; 3) multisectoral actions; 4) participatory processes; 5) equity toward universal health; 6) inclusion of traditionally excluded populations; 7) disaggregated data and targets; 8) monitoring; 9) accountability; and, 10) capacity to respond to health inequities. A full set of the questions and indicators is included in Table 1. The domains follow the conceptualization described above that centers both process and outcomes and that span the policy cycle—from developing the plan through key domains of its content, monitoring and evaluation, accountability, and further research to improve policies. Following Creswell and Poth (22), the rubric was verified through the reinforcing approaches of peer review and informant views; the former of which was accomplished through a small advisory group of experts from PAHO and Johns Hopkins University, and the latter occurred through a series of publications and a webinar with several hundred participants conducted in January 2019. An initial application of the rubric in two countries yielded further insights and slight changes to the rubric.

With the support of PAHO country offices and staff, the most recent written national health sector policies, strategies, and plans (NHPSPs) for countries in the Region of the Americas were collected. The World Health Organization (WHO) has urged all countries to create coherent NHPSPs—a distinct type of national policy document—arguing that “… strategizing – meaning designing plans and policies to achieve a particular goal related to the health of a nation – is absolutely critical in the 21st century” (8). These plans, as described by WHO, should be intersectoral and address both health and health equity within the overall national health planning process. Most countries in the Americas have an official NHPSP produced by government, and PAHO offices were able to share them upon request or verify if a country did not have one in place. NHPSPs were gathered for 32 countries of the Americas (see Annex 1 in supplementary material). The text of these plans formed the basis of the analysis in this paper.

Canada, Cuba, and the United States of America were not included in this analysis because, at the time of review (December 2019), these countries did not have a single national health plan, comparable to other countries in the Americas, that could be coded.^1^ Alternatives such as national health legislation are not comparable to national plans (for example, being narrowly focused on health insurance), and thus results could not be meaningfully compared with other countries.

While plans reviewed certainly do not represent a complete picture of the countries’ health policies, they do represent a perspective on the goals, intentions, and approaches national governments are taking within the health system at a given moment. Therefore, this article provides an initial foray into coding and analysis of health equity policies and should be viewed in that light.

Each of the gathered plans was coded on each indicator for inclusion in the plan or strategy, using a binary 0 or 1 coding on whether each factor was present in the plan. Sub-questions received fractional scores so that each question’s total was 1 (see Table 1). Sub-questions related to which populations health plans addressed were not assigned a score because the appropriateness of whether a particular population is included is context-dependent, but are reported below.

## RESULTS

### Overall findings: cross-national variation

The degree to which countries had incorporated equity into their national health plans varied considerably, as shown in Table 2, which offers a tabulation of the portion of questions in the rubric that the countries answered positively, signaling inclusion of policies to advance health equity. Overall, El Salvador, Colombia, Uruguay, Chile, and Honduras included the most elements from the rubric, and no country included all parts of the rubric, with the highest score being 22 out of 31 indicators coded “yes.” However, apart from the questions about recruiting underrepresented people into the workforce and financing models for social determinants of health, at least a handful of countries received a “yes” for each question.

**TABLE 1.** Health equity indicators rubric

Main topic		Indicators	Question score	Section score
**1. Mission**		A. Is health equity included as part of the health plan’s mission or vision? (Y/N)	1	1
**2. Social and environmental determinants of health**		i. Does the health plan incorporate measures to improve underlying determinants of health (e.g., increasing access to nutritious food, safe water, improved sanitation, healthier environments)?	1	3
		ii. Does the plan include financing models to incentivize health sector action on the social determinants of health?	1	
		iii. Does the plan include actions that the health sector is taking to respond to climate change?	1	
**3. Multisectoral actions**		i. Does the health plan include any measures to address health equity in the private sector?	1	1
**4. Participatory processes**	A. Were any participatory processes/mechanisms used to develop the national health plan?	i. Does the health plan refer to or describe a process in developing the plan that included public engagement, civil society engagement, or both?	1	5
		ii. If yes to 3Ai, does the plan refer to specific outreach to or inclusion of populations in situations of vulnerability?	1	
		iii. If yes 3Ai, did the process refer to the participation of non-health sectors in developing the plan?	1	
	B. Does the national health plan include any participatory processes/mechanisms for developing and implementing health policies and programs?	i. Does the health plan refer to the importance of public participation in developing and implementing and refer to specific mechanisms for public (or civil society) participation?	1	
		ii. If yes to 3Bi, does the health plan include any actions to support the functioning of these mechanisms (e.g., funding, training, outreach to populations in situations of vulnerability)?	1	
**5. Equity toward universal health**	A. Does the national health plan include actions towards achieving equity within the health sector?	i. Non-discrimination: 1. Does the health plan incorporate or refer to a strategy to address discrimination in the health sector?	1	7
		ii. Physical accessibility 1. Does the health plan include at least one action (other than health workforce related) to increase accessibility to quality primary health services in remote, rural, or otherwise underserved geographic areas or communities (e.g., constructing facilities in these areas, mobile health clinics, telemedicine)? 2. Does the health plan include at least one action to ensure the accessibility of health facilities for people with disabilities?	0.5 (ii.1) 0.5 (ii.2)	
		iii. Health workforce 1. Does the health plan include actions to increase the number of health workers in underserved communities to the health workforce? 2. Does the health plan include any actions regarding recruiting people from underrepresented communities into the health workforce, including management or other positions of authority?	0.5 (iii.1) 0.5 (iii.2)	
		iv. Health financing 1. Does the health plan include interventions to increase health service affordability for disadvantaged populations (e.g., delinking health service use from costs for these populations, subsidies)? 2. Does the health plan include strategies to increase the equitable distribution of health funding (e.g., more funding to communities with worse health outcomes, more disadvantaged populations)?	0.5 (iv.1) 0.5 (iv.2)	
		v. Health information 1. Does the health plan include any actions to increase health literacy of marginalized populations? 2. Does the health plan address language barriers to health services (e.g., interpretation services, health workforce recruitment from linguistic minorities)?	0.5 (v.1) 0.5 (v.2)	
		vi. Medicines and medical technologies (stock-outs/supply chain underserved areas, affordability) Does the health plan include interventions to increase access of marginalized populations to medicines (e.g., addressing affordability, improving supply chains to reduce stock-outs in remote areas)?	1	
		B. Does the health plan include a goal of universal health coverage?	1	
**6. Inclusion of marginalized populations**	A. Does the national health plan consider specific marginalized populations?	i. Does the health plan identify specific marginalized populations who face extra obstacles to equal health? (Y/N) 1. Are Afro-descendants among the populations identified? i. Does the health plan identify specific marginalized populations who face extra obstacles to equal health? (Y/N) 2. Are Indigenous peoples among the populations identified? i. Does the health plan identify specific marginalized populations who face extra obstacles to equal health? (Y/N) 3. Are Roma peoples among the populations identified? i. Does the health plan identify specific marginalized populations who face extra obstacles to equal health? (Y/N) 4. Are people with disabilities among the populations identified? i. Does the health plan identify specific marginalized populations who face extra obstacles to equal health? (Y/N) 5. Are members of the LGBTI community among the populations identified? i. Does the health plan identify specific marginalized populations who face extra obstacles to equal health? (Y/N) 6. Are migrants among the populations identified? i. Does the health plan identify specific marginalized populations who face extra obstacles to equal health? (Y/N) 7. Are people living in situations of poverty among the populations identified? i. Does the health plan identify specific marginalized populations who face extra obstacles to equal health? (Y/N) 8. Are other populations living in situation of vulnerability according to the national context among the populations identified?	1 (Note: Point given for identifying population at all, noted but not scored for which groups are included since different countries have different mixes of groups appropriate to identify)	3
		ii. Does the health plan includes specific actions to reduce barriers to good health for identified marginalized populations?	1	
		iii. Does the health plan refer to any actions to ensure that programs and services are differentiated to meet distinct needs of women, girls, men, and boys?	1	
**7. Disaggregated data and targets**	A. Does the health plan include collection of disaggregated data and use this data to set targets?	i. Does the plan include baseline data on health inequities across multiple dimensions (e.g., income, gender, age, race, ethnicity, indigenous status, migratory status, disability, geographic location)?	1	3
		ii. If disaggregated data is included, does the health plan include data disaggregated by the dimensions included in target 17.18 of the Sustainable Development Goals (income, gender [sex], age, race, ethnicity, migratory status, disability, geographic location, and other characteristics relevant in national contexts)?	1	
		iii. Does the health plan includes time-bound targets on reducing absolute or relative health inequalities in health service access (coverage) or in health outcomes?	1	
**8. Monitoring**	A. Does the health plan include processes for monitoring progress in its implementation?	i. Does the health plan include any process for regularly monitoring and evaluating its objectives and targets?	1	3
		ii. Does the health plan made readily accessible to the public? 1. Is the health plan available online? 2. Does the health plan include any strategies for communicating the plan’s contents to the public including members of marginalized communities?	0.5 (ii.1) 0.5 (ii.2)	
		iii. Does the health plan include a role for the public in monitoring and evaluating the health plan’s implementation?	1	
**9. Accountability**	A. Does the health plan include mechanisms to redress violations of people’s right to health?	i. Does the health plan include mechanisms for educating people on their right to health?	1	4
		ii. Does the health plan include mechanisms for reporting right to health violations?	1	
		iii. Does the health plan include mechanisms for enforcing people’s right to health?	1	
		iv. Does the plan include mechanism for investigating and reducing fraud and corruption?	1	
**10. Capacity to respond to health inequities**	A. Does the health plan include any actions on research to better understand and address health inequities?		1	1
**Total**				31

***Source:*** Prepared by the authors based on the results of this study.

**TABLE 2.** Inclusion of equity in national health plans as scored against 31 indicators across 10 domains

	1. Mission	2. Social and environmental determinants of health	3. Multisectoral actions	4. Participatory processes	5. Equity toward universal health	6. Inclusion of marginalized populations	7. Disaggregated data and targets	8. Monitoring	9. Accountability	10. Capacity to respond to health inequities		Total score/ranking		
**Max. value/score**	1	3	1	5	7	3	3	3	4	1		31	100%	
Antigua and Barbuda	1	2	1	2	2	2	0	1.5	0	0		11.5		**37.1**
Argentina	1	1	0	1	1	0	0	1	0	0		5		**16.1**
Bahamas	1	2	0	2	3	1	0	2 .5	1	1		13.5		**43.5**
Barbados	1	2	0	2	1	1	0	0.5	0	0		7.5		**24.2**
Belize	1	0	1	3	2	1	3	2 .5	0	1		14.5		**46.8**
Bolivia	1	2	0	3	4.5	2	2	1.5	1	0		17		**54.8**
Brazil	1	1	0	2	4.5	2	0	2 .5	2	0		15		**48.4**
Chile	1	2	0	4	4	2	3	3	0	0		19		**61.3**
Colombia	1	2	0	5	4	3	2	2.5	0	0		19.5		**62.9**
Costa Rica	1	2	0	2	2	1	1	2	1	0		12		**38.7**
Dominica	1	1	0	3	2.5	2	2	2.5	0	0		14		**45.2**
Dominican Republic	1	1	0	0	1.5	2	2	2	0	0		9.5		**30.6**
Ecuador	1	1	0	3	2.5	2	2	2	0	0		13.5		**43.5**
El Salvador	1	2	1	3	5.5	3	0	2.5	3	1		22		**71.0**
Grenada	1	2	0	3	3	3	1	0.5	0	0		13.5		**43.5**
Guatemala	1	1	0	1	1.5	2	0	1.5	0	0		8		**25.8**
Guyana	1	1	1	4	5	2	0	3	0	0		17		**54.8**
Haiti	1	1	1	2	3	3	1	1.5	1	1		15.5		**50.0**
Honduras	1	2	1	3	4 .5	3	2	1.5	1	0		19		**61.3**
Jamaica	1	2	1	3	3 .5	2	2	1.5	0	0		16		**51.6**
Mexico	1	1	0	0	2.5	2	1	1.5	0	0		9		**29.0**
Nicaragua	1	1	0	1	1.5	2	0	1.5	1	0		9		**29.0**
Panama	1	2	0	1	2.5	2	2	3	1	1		15.5		**50.0**
Paraguay	1	2	0	3	2.5	1	0	0.5	0	0		10		**32.3**
Peru	1	2	0	2	4 .5	2	2	2.5	0	0		16		**51.6**
Saint Kitts and Nevis	1	1	1	3	3 .5	2	3	1	0	1		16.5		**53.2**
Saint Lucia	1	2	0	2	0	0	0	1.5	1	0		7.5		**24.2**
Saint Vincent and the Grenadines	0	2	0	0	0.5	0	1	1	0	0		4.5		**14.5**
Suriname	1	2	1	2	2.5	1	3	1.5	1	1		16		**51.6**
Trinidad and Tobago	1	1	1	1	1	0	1	2	0	0		8		**25.8**
Uruguay	1	1	1	3	5.5	3	2	2	1	0		19.5		**62.9**
Venezuela	0	0	0	1	3	1	1	0	0	0		6		**19.4**
											**Full score**			**31**
											**Average score**			**13.13**
											**Min score**			**4.5**
											**Max score**			**22**

***Source:*** Prepared by the authors based on the results of this study.

**FIGURE 1.**
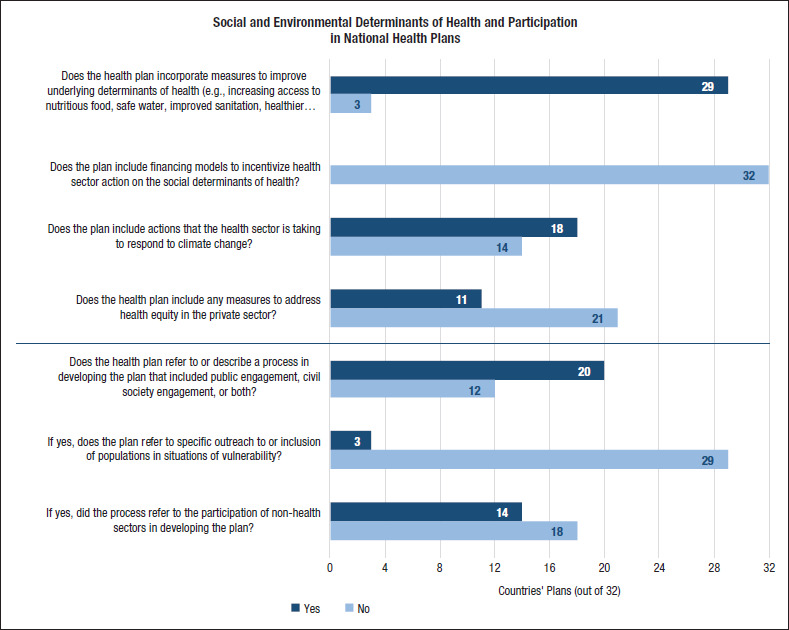
Inclusion of social and environmental determinants of health and participation in 32 national health plans

***Source:*** Prepared by the authors based on the results of this study.

How countries incorporated equity varied considerably. For example, Chile, Colombia, and Guyana included many of the indicators of participation in their plans, while countries including the Dominican Republic, Mexico, and Saint Vincent and the Grenadines did so to a lesser extent. Brazil, El Salvador, and Honduras focused less on participation in their plans but included many of the universal health and health care elements. Unlike most countries, Belize, Chile, Saint Kitts and Nevis, and Suriname included all three of the elements coded for on disaggregating data and targets.

### Mission, vision, and social determinants of health

Almost all countries, 30 out of the 32, included health equity as part of their health plan’s mission or vision (or elsewhere in the document). Chile’s Estrategia Nacional de Salud para el Cumplimiento de los Objetivos Sanitarios 2011–2020 provides an example, with its fifth Strategic Objective being “to reduce health inequities of the population by mitigating the effects produced by social and economic determinants of health.”

Likewise, 30 of 32 of the countries’ health plans address underlying determinants of health, such as increasing access to nutritious food, safe water, improved sanitation, or healthier environments (Figure 1). Barbados’s national health plan exemplifies an affirmative answer to this question. Its plan includes actions related to food and nutrition and access to water and sanitation. It also includes specific targets for this objective, with a commitment to a 50% reduction in dependence on food imports and to develop a National Food Security Program by 2010.

In contrast to the frequent positive findings for measures to improve underlying determinants of health, no country had financing models that incentivize addressing the social determinants of health. However, Belize’s Health Sector Strategic Plan 2014–2024 addresses road safety financing, mentioning a financing mechanism for a road safety project funded by the Inter-American Development Bank.

Several other areas in the domain of social determinants of health provided a more mixed picture. Just over half (18 of 32) of the analyzed countries’ plans include measures to respond to climate change. Meanwhile, despite the private sector playing a growing role in many countries, only 11 out of 32 countries addressed health equity in the private sector in their health plans.

### Participation in plan design and implementation

More than half of the analyzed country plans (20/32) describe a process for developing the plan that included public engagement, civil society engagement, or both (Figure 1). For example, Colombia’s Plan Decenal de Salud Pública 2012–2021 was developed with public consultation during the plan design process. However, few health plans refer to outreach to specific marginalized (or other) populations.

Encouragingly, most national health plans recognize the need for public participation and refer to specific mechanisms for public (or civil society) participation in developing and implementing policies and programs (28 of 32 countries). Guyana’s National Health Policy is exemplary. It includes additional guiding principles dedicated to “active social participation” and incorporates a National Health Policy Committee as a mechanism to include the “civil society and private sector organizations” in strengthening “the legislative, institutional, and policy framework of the health system.”

However, there were few references to these participatory mechanisms being funded, structured efforts. Brazil is one of the 5 (out of 32) countries that include any actions to support the functioning of these mechanisms through, for example, supporting the establishment of decentralized ombudsman structures, implementing policies to encourage the evaluation of services by users, and disseminating information about the right to health and the exercise of such a right.

### Equity toward universal health and health care

Substantively, plans showed significant diversity in how they addressed health and health care overall, including measures that are key to health equity. Most plans (23) include a goal to provide universal health coverage. Specific steps toward equity in ensuring health care for all, however, were less common. The most common areas addressed by the plans on this front were medicines—with just under half of plans including interventions to increase access of marginalized populations to medicines (e.g., addressing affordability, reducing stock-outs in remote areas)—and physical accessibility, with just over half of plans including at least one action to increase accessibility to quality primary health services in remote, rural, or otherwise underserved geographic areas or communities.

Ten of 32 countries included actions to increase the number of health workers in underserved communities, though only Jamaica included measures on recruiting people from underrepresented communities into the health workforce. A similar number include interventions to increase health service affordability for disadvantaged populations (14 countries) as include interventions to increase the equitable distribution of health funding (13 countries).

### Discrimination and identification of populations in situations of vulnerability

Eleven out of 32 countries’ health plans “incorporate or refer to a strategy to address discrimination in the health sector.” Costa Rica’s health plan offers a good example, with two strategies to be applied across the health sector to address gender inequality and violence against LGBT people.

**FIGURE 2.**
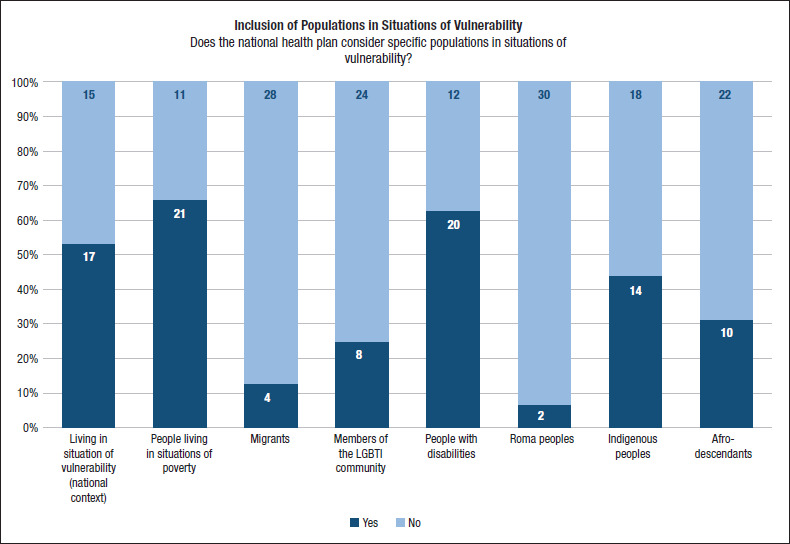
Inclusion of populations in situations of vulnerability in 32 national health plans

***Source:*** Prepared by the authors based on the results of this study.

Despite the small number of health plans that include non-discrimination strategies, most plans reviewed did identify multiple populations that face obstacles to equal health. Figure 2 shows the number of countries listing each of eight different population categories in their national plan. People living in poverty and people with disabilities are the two socially excluded groups most often mentioned in the health plans. Roma peoples and migrants are the least mentioned. Recognizing that it might be quite reasonable for some countries that are not home to certain populations—for example, many simply might not have a Roma population—the inclusion of these populations was assessed, but did not factor into scoring. Whether an exclusion is well-justified or an omission that should be rectified requires an assessment of the country context that was beyond the scope of this study.

### Data, monitoring, and accountability

More than half of the analyzed country plans (19/32) include baseline data on health inequities across multiple dimensions (e.g., income, gender, age, race, ethnicity, migratory status, disability, geographic location). Forty-one percent of the plans (13/32) have time-bound targets on reducing absolute or relative health inequalities in health service access or health outcomes. Panama is a good example; its Política Nacional de Salud y Lineamientos Estratégicos 2016–2025 analyzes the country’s health situation with disaggregated data on several health-related issues.

Fewer national development strategies—which were also analyzed along with national health plans for a small set of indicators—include time-bound indicators or targets for health equity. Almost half of countries (15 of 32) include specific indicators and time-bound targets (sometimes still to be developed) for health overall, though only about half of these countries (8 countries) include one or more indicators or targets related to equity. Of the eight countries that include health equity targets in their national development strategies, six also have such targets in their national health plans (of the 13 total national health plans with such targets).

Eighty-four percent of the national health plans (27 out of 32) incorporate a process for regularly monitoring and evaluating their objectives and targets. However, only 31% (10/32 countries) include a role for the public in monitoring and assessing the health plan’s implementation. The degree of specificity of the monitoring and evaluation processes varies. For example, Honduras includes a general description of the monitoring process, and Suriname’s health plan provides monitoring as an objective of the plan itself and specific targets to fulfill it, together with creating two monitoring and implementation committees.

Very few countries’ health plans addressed accountability mechanisms tied to the right to health. Only two countries’ health plans discuss mechanisms for reporting violations to the right of health, and only three mention mechanisms for investigating and reducing fraud and corruption.

## DISCUSSION

Setting out to identify whether countries in the Americas are planning to address health equity, this study shows mixed results, with significant reason to believe that planning could be strengthened. The PAHO Equity Commission’s recommendations are anchored in governance shifts that start with a call to “develop strategic plans for improving health equity” (19). Reviewing national health sector plans shows that this is indeed a gap for all countries in the region, even as many have taken up key pieces of this work already and could share their experiences between countries.

The PAHO Equity Commission’s recommendations are not yet embodied in the current health sector plans of the Americas. However, there is reason for optimism, as countries *are* giving attention to health equity in their written national health sector policies, strategies, and plans. Nearly all explicitly include health equity as a clear goal of these plans.

As recommended by PAHO, the overwhelming majority include specific attention on the social determinants of health in their plans. With a growing role of the private sector in many countries in the region, with significant implications for equity, it is notable that few plans address that sector.

Attention to participation varies greatly among countries; one country includes all of the indicators, a handful of countries include many of them, and some include none.

No country includes all measures of substantive equity in health systems, but quite a few include several of these measures, such as improving physical and financial accessibility, and increasing access to medicines for socially excluded populations. Fewer countries addressed other areas, including addressing discrimination, increasing access to health workers in underserved areas, and removing language barriers. On the whole, countries include attention to socially excluded populations in their health plans, though with a few notable limitations: only about a quarter of plans identify Afro-descendants; fewer than half identify Indigenous people, with some countries with large Indigenous populations not addressing them. Only four countries in the region include attention to migrants.

A minority of countries, only 41%, include time-bound targets on reducing absolute or relative health inequalities. Interestingly, the setting of time-bound targets corresponds fairly often with countries with equity-robust plans across the board. However, there are exceptions, including Mexico, which sets clear targets for health equity, even though the country includes only 10 out of 31 indicators in the national health plan. Like El Salvador, a few countries with the most robust plans have not yet set time-bound equity targets.

Overall, very few countries included clear accountability mechanisms that we might hope to see in plans addressing health equity, with just a handful including references to education, reporting, or enforcement mechanisms in this area.

It is noteworthy that of the 32 countries whose health plans we reviewed, the average score under this rubric was inclusion of just 13 out of 31 indicators in their plans; no country includes more than about 70%. There is work to do in planning throughout the region. It is notable too that some countries with better health outcomes, like Argentina, pay relatively little attention to health equity in their national health plans; while some like Haiti, with the region’s lowest life expectancy, have more robust attention in their plans. There is, of course, no simple causal line between the content of written plans, which is the narrow focus of this study, and health outcomes. Yet this study does tell us something about a starting point for addressing health inequalities—which remain urgent in both Argentina and Haiti. Measuring the problem, setting targets for progress, and building mechanisms of accountability are all widely recognized tools in effective planning—tools which these data show are underutilized in addressing health equity.

This analysis has several limitations. First, there is an inherent limitation in seeking to understand a country’s policy environment and actions to advance health equity through reviewing documented plans, both because other laws and policies affect health equity and because countries’ health and development plans can only be fully understood in countries’ overall political, institutional, and social contexts, including progress already made toward greater health equity. Therefore. this study’s modest goals should not be over-interpreted. Future analysis of a broader set of legal and policy documents could prove fruitful in expanding the picture. In addition, countries take different approaches to the level of specificity and granularity, reflecting that these findings are influenced by the broader characteristics of the planning process and documentation in a given country. That said, the animating theory behind this work is that measuring, planning, and creating accountability can be important for improving health equity, and these findings provide an initial representation of national attention to those factors.

### Conclusion

The nearly unanimous commitment across countries of the Americas to equity in health, as expressed in their national health plans, provides an important opportunity to advance the agenda of addressing inequity. We find, however, significant variation in the substance and structure of how health plans in the Americas handle the issue.

It is helpful that, in many countries, baseline data are available in national plans on several axes of inequality, against which progress could be judged. In other countries, such baseline data will be necessary for real planning to help address equity. Political will to translate goals into impact will be seen in the coming years in whether time-bound targets are set and achieved. So far, fewer than half of countries include time-bound targets on reducing absolute or relative health inequalities, which is likely to undermine progress on equity.

Substantive equity-focused policies, such as those to improve physical accessibility of health care and increase affordable access to medicines, are included in many plans, though no country includes all aspects examined. Participatory processes documented in the development of these plans range from none to extensive and robust vis-à-vis the policies, target-setting, and accountability included in health plans. This suggests that both technical support on health equity when plans are crafted and peer-learning could be beneficial in supporting planning to reach stated goals. Further, there remain gaps in identifying actions to address inequities among marginalized populations—particularly Afro-descendants, Indigenous people, and migrants.

The diversity of plans presents a strong opportunity for learning. Insofar as some countries have created detailed, equity-robust plans, this might provide ideas. But no two plans are the same, and even the countries with more robust plans could take inspiration from others.

The rubric developed in this study represents a step toward assessing and understanding the policy environment for health equity that could be applied, in future work, to a wider range of health policies, laws, and strategies. This can be helpful, too, in future work to understand what kinds of policies are particularly effective and support regional learning. Policy-making is an intervention—aimed at taking ideas to a national scale—and subjecting it to review, evaluation, and improvement can only help achieve the widespread ambition of reducing inequalities across the region.

## Disclaimer.

Authors hold sole responsibility for the views expressed in the manuscript, which may not necessarily reflect the opinion or policy of the *RPSP/PAJPH* and/or PAHO.

## Footnotes

1 A health equity analysis of the United States of America’s Affordable Care Act (ACA) was conducted by Grogan in 2017 (23). However, because the ACA is a piece of health legislation with decentralized implementation, rather than a national health plan, and primarily (though not exclusively) focused on healthcare financing, especially health insurance, it is unsuitable for inclusion in this comparison.
